# Defining the Profile of Patients with Primary Merkel Cell Carcinoma in Central Italy: A Retrospective Analysis

**DOI:** 10.3390/cancers14205140

**Published:** 2022-10-20

**Authors:** Giulio Gualdi, Gerardo Ferrara, Marco Simonacci, Maria Concetta Fargnoli, Laura Sollima, Elisa Molinelli, Anna Campanati, Giampiero Mazzocchetti, Alfredo Giacchetti, Paolo Amerio, on behalf of the Study Group

**Affiliations:** 1 Department of Medicine and Aging Science, Dermatologic Clinic, University G. D’Annunzio, Chieti Pescara, 66100 Chieti, Italy; 2 Anatomic Pathology and Cytopathology Unit, Istituto Nazionale Tumori IRCCS Fondazione ‘G. Pascale’, 80131 Naples, Italy; 3 Department of Dermatology, Macerata Hospital, 62100 Macerata, Italy; 4 Department of Biotechnological and Applied Clinical Sciences, University of L’Aquila, 67100 L’Aquila, Italy; 5 UOC Pathology, Ospedale San Salvatore, 67100 L’Aquila, Italy; 6 Department of Clinical and Molecular Sciences—Dermatological Clinic, Polytechnic Marche University, 60121 Ancona, Italy; 7 UOSD Dermatologia ASL1 Pescara, 65124 Pescara, Italy; 8 UO Dermatologia, INRCA/IRCCS, 60129 Ancona, Italy

**Keywords:** epidemiology, dermatology, Italy, Merkel Cell Carcinoma, observational study, retrospective analysis

## Abstract

**Simple Summary:**

Merkel cell carcinoma is rarely suspected and often misdiagnosed in the clinical setting. Robust epidemiological data are missing, contributing to a lack of knowledge of this type of tumor among clinicians. We aimed to demographically and clinically characterize Merkel cell carcinoma patients and the tumor’s features through a multicenter real-world analysis of patients from Central Italy from 2015 to 2020. Our study revealed a higher incidence rate compared to the one estimated for the Italian population, with the lower limbs as the anatomical site affected the most. We also report that several specialists are involved in the first-line management of the pathology. In this light, a deeper knowledge of this tumor is advised.

**Abstract:**

Merkel cell carcinoma (MCC) is a rare neuroendocrine skin cancer that usually occurs in elderly people on sun-exposed areas, with a predisposition to local recurrence. Evidence suggests a growing incidence over the past decade; however, robust epidemiologic data are still lacking. We describe the MCC population in clinical practice in a retrospective analysis of demographic, clinical, and tumor characteristics from medical records of primary MCC patients, between 2015 and 2020, at six dermatology clinics in Central Italy. Ninety-four patients were included (57.4% male; mean age 78.2 ± 10.1 years, range 47–99 years). The estimated incidence rate of MCC was 0.93 per 100,000 inhabitants/year. Lower limbs were the most frequently affected site (31.5%), and 54% of patients for whom information was available were immunosuppressed. Lymph node involvement was reported in 42.5% of patients, and distant metastases in almost 20%. Most patients underwent surgery for tumor excision and were mainly referred to specialized dermatology clinics by dermatologists (47.9%) and general surgeons (28.7%). Apart from the relatively balanced prevalence of MCC in men and women, the predominant location on lower limbs, and the higher incidence rate compared with previous reports in Italy, this population is, overall, similar to the populations described in other observational studies. MCC management requires the involvement of several specialties. Increased awareness of MCC and standardization of its management are urgently needed.

## 1. Introduction

Merkel Cell Carcinoma (MCC) is a rare and aggressive neuroendocrine skin cancer [1,2,3]. Although accounting for less than 1% of all cutaneous malignancies [4], MCC ranks among the most lethal, with an approximately two-fold higher mortality rate than melanoma [5]. The epidemiology of MCC is not well defined, but data from several countries suggest a steadily growing incidence [6,7,8,9]. The exact etiology of MCC is still debated [2,4]. Exposure to ultraviolet (UV) light and cell transformation by a polyoma virus, the so-called Merkel cell polyoma virus (MCPyV), have been suggested by a large body of evidence as causative factors [1,5,10,11].

The clinical presentation of MCC is effectively summarized by the acronym AEIOU, where A stands for “asymptomatic/lack of tenderness”, E for “expanding rapidly”, I for “immune suppression”, O for “older than age 50”, and U for “UV-exposed site in a person with fair skin” [12]. Specifically, MCC generally appears as an asymptomatic pink-to-dull red nodule on sun-exposed body parts. On average, the diagnosis is performed on patients over the age of 70, in which a history of immunosuppression is frequently reported [1]. Early diagnosis is crucial, as MCC can be treated effectively in its early stages [1].

Treatment traditionally includes surgery (first-line option, when feasible), radiotherapy, and chemotherapy [1]. Patients should be referred to high-volume specialized centers for adequate management [13], and the involvement of a multidisciplinary team is recommended by current guidelines [1,3]. In recent years, promising results have been achieved in the development of cancer immunotherapy, which led to the approval of the PD-1/PD-L1 immune checkpoint inhibitor avelumab [14] (approved by the US Food and Drug Administration (FDA) and the European Medicine Agency (EMA)).

Despite increased treatment options and the improvement in disease detection by using biomarkers [1,15], MCC management continues to be challenging in routine dermatologic practice. Due to its rarity and lack of specific symptoms, MCC is often unrecognized and misdiagnosed as a benign lesion [1,12]. Although international practical guidelines specifically devoted to MCC are available [1,16], recommendations are often ignored [17,18]. Furthermore, there is a lack of unified and shared protocols in clinical practice, and MCC patients are often treated by different specialists, which contributes to the loss of information. Ideally, the quality and consistency of data collection from MCC patients should be encouraged and improved. 

In order to profile the MCC population typically encountered in clinical practice, we describe here the characteristics of patients diagnosed with primary MCC in six centers in Central Italy between 2015 and 2020. 

## 2. Patients and Methods

This study was a multicenter, retrospective analysis of medical records from patients diagnosed with primary MCC between 1 January 2015 and 31 December 2020, at the dermatology and pathology departments of six referral clinics in two Central regions of Italy (Marche and Abruzzo). Given its observational design, the study does not require a formal patient consent, as well as Ethic Committee approval, according to the Italian law [19]. All patients involved in the analysis lived in the area and had their diagnosis confirmed by revision of the histopathological examination. The following data, recorded at presentation and during the diagnostic procedure, were extracted from the patient’s charts: demographic characteristics; medical history (with particular attention to immunosuppression); tumor characteristics including anatomic location, tumor size and extent (the T of the TNM staging system; TX, the primary tumor cannot be assessed; T0, no evidence of primary tumor; Tis, in situ primary tumor; T1, maximum clinical tumor diameter ≤2 cm; T2, >2 cm but ≤5 cm; T3, >5 cm; T4, primary tumor invades other tissues) [1], tumor thickness, mitotic rate, presence of ulceration, number of affected regional lymph nodes, and presence of distant metastases. The types of surgeries performed and the referring specialists were also recorded. Data were summarized by descriptive statistics. An estimate of the incidence of MCC was calculated based on the demographic data of the studied areas [20].

All patients gave informed consent for participation in the study. The study was conducted according to the Declaration of Helsinki.

## 3. Results

Overall, 94 patients with a confirmed diagnosis of MCC were included in the analysis (Table 1), with an estimated incidence rate of 0.93 per 100,000 inhabitants per year calculated. There was a slightly higher percentage of men than women in the patient population (57.4% vs. 42.6%), and the mean age was 78.2 ± 10.1 years (range 47–99 years), with 83% of patients aged ≥70 years. Thirteen patients (54.2%) were reported to be immunosuppressed and eleven (45.8%) immunocompetent (information about immunosuppression was not available for 70 patients). 

The most commonly affected body parts were the lower limbs (31.5%), with a slightly greater prevalence in women (59% vs. 41%), head and face (27.2%), and upper limbs (14.1%) (Table 2). Ten patients (10.9%) presented with primary involvement of lymph nodes, with no skin manifestation of MCC. 

**Figure 1:**
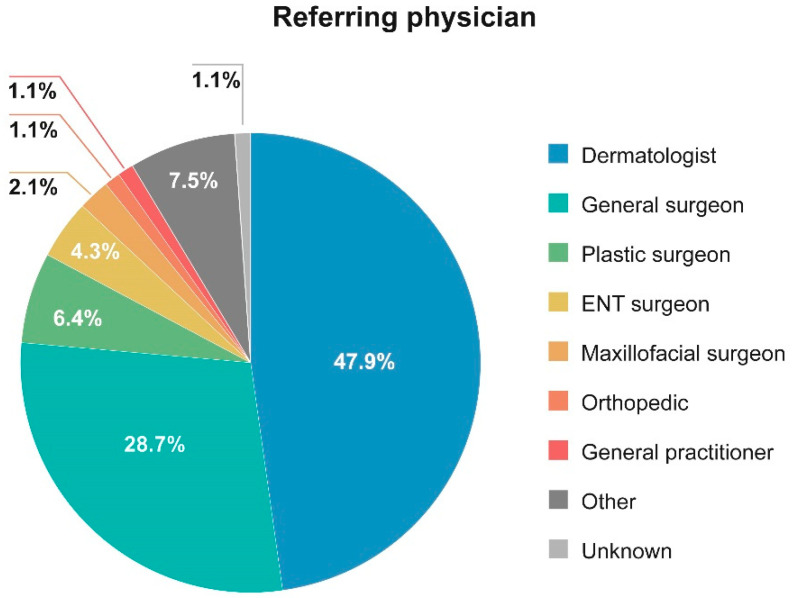
Distribution of the physicians who referred patients to the centers.

Most patients were referred to the study centers by a dermatologist (47.9%) or a general surgeon (28.7%) (Figure 1). 

In the majority of the patients with a measurable tumor and available T data, the maximum clinical tumor diameter was ≤2 cm (corresponding to T1) (Table 3). The mean tumor thickness was 8.1 mm (range: 0.7–26 mm); on average, patients had a mitotic rate of 27.2 mitotic figures per mm^2^. Among the patients for whom the chart information on the presence/absence of skin ulceration at the MCC site was available (*n* = 66), 28 (42.4%) had ulcerations. During the development of the disease, lymph node involvement and distant metastases were reported in 42.5% and 20% of patients, respectively. Most patients (74/92, 80.4%) underwent surgery for tumor excision (of these, 13.5% (10/74) had affected margins), and the remaining patients (18/92, 19.6%) were subjected to a diagnostic biopsy. 

## 4. Discussion

The present study describes the demographic, clinical, and tumor characteristics of patients that were diagnosed with MCC and referred to six dermatology clinics in Italy between 2015 and 2020. The results obtained are in line with the profile of MCC patients reported in other studies [2,5], as shown by the advanced mean age (>75 years) and high prevalence of locoregional metastases. However, in contrast with the published data suggesting a marked predominance in men [4], we report a relatively balanced prevalence of MCC in men and women. In addition, our study differed from published reports with regard to the most common anatomical localization of MCC skin lesions. In our patient population, lower limbs were the most frequent MCC site reported, mainly in women, whereas other studies have reported that the head and neck are usually more affected [2]. The interpretation of these observations is currently unclear to us. It is also unclear whether MCPyV+ MCC and UV-related MCC occur at the same sites or have a distinct anatomical localization pattern [2]. Another interesting factor is the relatively high nodal localization with no apparent evidence of primary cutaneous tumor, which discloses two possibilities: a complete regression of the skin tumor or a primary nodal localization. This observation could be relevant in terms of the adjuvant therapy definition.

Patients in this study were mainly referred to specialized dermatology clinics by dermatologists and general surgeons and, to some extent, plastic surgeons. This highlights the relatively broad spectrum of specialists involved in the initial management of MCC in clinical practice and suggests that future efforts to increase awareness of this rare and aggressive skin cancer should be directed to general practitioners, dermatologists, general surgeons, and plastic surgeons in order to improve their ability to diagnose MCC. However, more data are necessary to evaluate how the different specialists manage MCC. 

The estimated annual MCC incidence rate of 0.93 per 100,000 inhabitants is higher than the one estimated by the Associazione Italiana Registri Tumori (AIRTUM) [21]. According to the AIRTUM, the annual incidence rate of neuroendocrine skin carcinoma in Italy was 0.34 per 100,000 inhabitants (2000–2010), with 238 new cases estimated in 2015 [21]. The higher incidence reported here may be due to the limited population screened and the minor reduction in the number of inhabitants in the studied area between 2015 and 2020. Another explanation for this difference may come from the greater sun exposure, due to climate factors, in our population compared to the average exposure of the Italian population. Indeed, the population of Central regions of Italy is distributed mainly on the coast, the mean age is higher than the general Italian population, and a high percentage is still engaged in rural activities [20]. Higher incidence rates, closer to the one estimated in the present study, have been reported in other countries [7,8,9]. For example, Paulson et al. in 2013 estimated an MCC incidence rate of 0.7 per 100,000 person-years in the US, corresponding to 2488 cases [7], and an analysis of an Australian registry of 1095 MCC cases diagnosed between 1986 and 2016 estimated an incidence rate of 3.9 per 100,000 in men and 1.5 per 100,000 in women [8]. 

Our analysis has several limitations: a retrospective design and the small size of the study population; indeed, a study involving other centers could be appropriate to evaluate the epidemiology of the pathology. The present study took advantage from the good network already established within these centers in Central Italy. We believe that our work adds more information to the known epidemiology and clinical characteristics of Merkel Cell Carcinoma in Italy, that until now was limited only to case series or single center studies. Moreover, there is a suggestion for a north-to-south gradient in MCC in Italy [22], and most of the papers published regarded Northern Italy centers. Our work contributes to expand the knowledge.

This is a retrospective study that analyzed the characteristics of MCC patients by coupling the pathological reports with clinical records of the different centers involved. Evaluation of the specific treatment was not an aim of the study as well as a detailed description of MCC. Data on chemotherapy were not available for all patients; nevertheless, they were treated following AIOM (Italian Association of Medical Oncology) guidelines [23], i.e., surgery or surgery and radiotherapy, with chemotherapy and immunotherapy according to the different stage of the disease. Almost half of the cases were referred by dermatologists, which are the front line for the treatment of the disease, given the possibility of dermoscopy for the diagnosis. The importance of the AEIOU acronym for the diagnosis is pivotal in recognizing early MCC.

Moreover, our analysis has revealed inconsistencies in data recording. Therefore, an urgent need in MCC management is the standardization of patient records and pathology reports.

## 5. Conclusions

MCC management is complex. However, recent advances in identifying this rare and aggressive endocrine skin cancer and the potential of cancer immunotherapy may significantly improve patient outcomes. Increased awareness of MCC and standardization of its management are urgently needed.

## Figures and Tables

**Table 1:** Patients’ characteristics at presentation (N = 94).

Gender	*n* (%)
Female	40 (43)
Male	54 (57)
Age years, mean ± SD (range)	78.2 ± 10.1 (47–99)
Age groups	*n* (%)
<70 years	16 (17)
70–84 years	52 (55.3)
≥85 years	26 (27.7)
Immunosuppression	*n* (%)
Pharmacologic	8 (8.5)
Disease-related	5 (5.3)
No immunosuppression	11 (11.7)
Unknown	70 (74.5)

**Table 2:** Anatomic location of MCC at presentation.

Anatomic Location	*n* (%) ^a^
Lower limbs	29 (31.5)
Head and face	25 (27.2)
Upper limbs	13 (14.1)
Lymph nodes	10 (10.9)
Hands	5 (5.4)
Scalp	3 (3.3)
Chest and abdomen	2 (2.2)
Eyelids	2 (2.2)
Neck	1 (1.1)
Back	1 (1.1)
Feet	1 (1.1)

^a^ N = 92, as data were missing for two patients.

**Table 3:** Tumors’ characteristics (N = 94).

T Stage	*n* (%)
TX	7 (7.5)
T1	32 (34)
T2	17 (18.1)
T3	5 (5.3)
T4	10 (10.6)
Unknown	13 (13.8)
Thickness mm, mean (range)	8.1 (0.9–26)
Presence of ulceration	*n* (%)
Yes	28 (29.8)
No	38 (40.4)
Unknown	27 (28.7)
Lymph node involvement	*n* (%)
Yes	40 (42.5)
No	37 (39.4)
Unknown	17 (18.1)
Distant metastases	*n* (%)
Yes	17 (18.1)
No	61 (64.9)
Unknown	16 (17)
Mitotic rate number of mitotic figures/mm^2^, mean (range)	27.2 (5–77)

## Data Availability

The data presented in this study are available on request from the corresponding author.
